# Rapid biodegradation of microplastics generated from bio-based thermoplastic polyurethane

**DOI:** 10.1038/s41598-024-56492-6

**Published:** 2024-03-12

**Authors:** Marco N. Allemann, Marissa Tessman, Jaysen Reindel, Gordon B. Scofield, Payton Evans, Robert S. Pomeroy, Michael D. Burkart, Stephen P. Mayfield, Ryan Simkovsky

**Affiliations:** 1 Algenesis Corporation, 11760 Sorrento Valley Rd. Suite J, San Diego, CA 92121 USA; 2 Department of Chemistry and Biochemistry, University of California, San Diego, 9500 Gilman Drive, La Jolla, CA 92093 USA; 3 Division of Biological Sciences, University of California, San Diego, 9500 Gilman Drive, La Jolla, CA 92093 USA

**Keywords:** Environmental impact, Sustainability, Biomineralization, Applied microbiology, Soil microbiology

## Abstract

The accumulation of microplastics in various ecosystems has now been well documented and recent evidence suggests detrimental effects on various biological processes due to this pollution. Accumulation of microplastics in the natural environment is ultimately due to the chemical nature of widely used petroleum-based plastic polymers, which typically are inaccessible to biological processing. One way to mitigate this crisis is adoption of plastics that biodegrade if released into natural environments. In this work, we generated microplastic particles from a bio-based, biodegradable thermoplastic polyurethane (TPU-FC1) and demonstrated their rapid biodegradation via direct visualization and respirometry. Furthermore, we isolated multiple bacterial strains capable of using TPU-FC1 as a sole carbon source and characterized their depolymerization products. To visualize biodegradation of TPU materials as real-world products, we generated TPU-coated cotton fabric and an injection molded phone case and documented biodegradation by direct visualization and scanning electron microscopy (SEM), both of which indicated clear structural degradation of these materials and significant biofilm formation.

## Introduction

One of the major consequences of ubiquitous plastic usage by humans is the generation of microplastics: tiny plastic particles that persist in the environment and have now been documented to spread though all parts of the planet^1–4^. While the reported size range for microplastics varies, they are generally defined as particles between 5 mm and 500 µm in size^2^. These particles can originate from any plastic source, including bottles, bags, and packaging, as well as synthetic textiles, tire wear, and microbeads from personal care products^2^. Microplastics are typically formed through a variety of physical and chemical processes, such as physical or chemical-induced fragmentation, abrasion, manufacturing processes, and UV degradation^5^. Microplastics have now been documented to accumulate in all physical environments, such as water bodies, soil, precipitation, and even dispersed in the air^1,3,4^. Due to their ubiquity, microplastics are now part of the food chain, and early evidence suggests the potential for significant harm to ecosystems, animals, and humans from these materials.

An attractive solution to mitigate the environmental impact of microplastics is to develop plastics that do not generate persistent microplastics as part of their normal life cycle^6^. Even plastics that are properly collected and recycled generate microplastics as part of the normal wear from everyday use or as a consequence of recycling or washing processes^2,4^. Thus, to prevent the accumulation of microplastics, new plastic materials must be developed that are completely biodegradable so that any particles generated from these products will quickly degrade in the environment. Biodegradation is the process by which microbes break down polymers into simpler molecules that can be used as a source of carbon to produce biomass. This requires that the polymer contains chemical bonds, most notably in the polymer's primary backbone structure, that are physically accessible to enzymes that naturally recognize these bonds as substrates, and that the underlying monomer molecules that are released through this enzymatic cleavage can be consumed by microorganisms. In natural environments, this process is typically performed by consortia of microbes, including bacteria and fungi, secreting hydrolytic enzymes^7^, which sever the polymer to release a variety of monomers and oligomers that can then be utilized as a carbon nutrient source by the microbes. Catabolism of these polymer-derived oligomers and monomers leads to the generation of organismal biomass and CO_2_ via respiration. While many biobased materials are derived from natural renewable sources, they can potentially persist in the environment due to their limited ability to biodegrade or due to chemical processing that make them inaccessible to biological cleavage, as occurs with the vulcanization of rubber. It is also possible to generate biodegradable materials from petroleum or other fossil resources, as biodegradation is a property of the material rather than a property of where that material was derived.

Polyurethanes are a class of polymers that are widely used in a variety of products, including foam cushions, adhesives, coatings, and synthetic fibers^8,9^. Polyurethanes have many desirable properties depending on their formulation, such as being lightweight, durable, and flexible. A polyurethane polymer is composed of repeating units of polyols and isocyanates. A urethane bond, also known as a carbamate bond, is generated when an alcohol on the polyol reacts with an isocyanate group. Carbamates are found and metabolized in nature, including in hemoglobin and RuBisCo, and hence are suitable to make biodegradable polymers. Polyols can be formulated in a variety of ways that impact their ability to biodegrade. For example, polyester polyols are formed through reactive condensation of diacids and diols to generate ester linkages. Polymers containing ester bonds have the potential to biodegrade due to the susceptibility of ester linkages to hydrolysis, which can occur by abiotic mechanisms or enzymatic means as a consequence of the evolution of diverse esterases in response to the ubiquity of ester bonds found in nature^6^. In contrast, polyether polyols, most typically derived from petroleum, are used extensively in the current plastics industry because the ether bonds in the polymer are not susceptible to biodegradation. Thus, polyether polyurethanes derived from polyether polyols can also be a source of microplastic pollution when they break down into small particles through fragmentation processes. However, a polyester polyurethane polymer containing ester and urethane linkages has the potential for rapid and complete biodegradation when hydrolytic enzyme activity is allowed to proceed on the polymer^9–13^. The process and rate of biodegradation is dependent upon both the chemical and physical properties of the polymer, as well as the environmental condition to which the polymer is exposed, so that biodegradation or abiotic hydrolysis can only occur under specific conditions. For example, polylactic acid (PLA), a polyester polymer, has ester bonds that are susceptible to hydrolysis under elevated temperatures present in industrial compositing facilities, but not in marine environments^14^. We reasoned that microplastics generated from environmentally biodegradable polyester polyurethanes might rapidly degrade in the environment and hence not persist in the natural environment.

**Figure 1:**
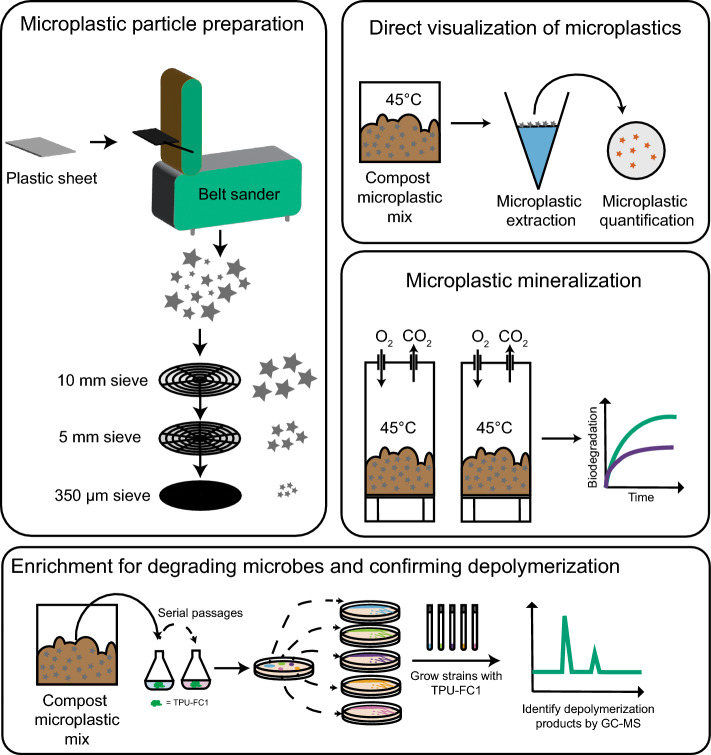
Generating microplastics particles and tracking their biodegradation in compost. Microplastic particles were generated by sanding solid sheets of plastic materials and size selection of the resulting particulates by sieving. Particles smaller than 5 mm and larger than 350 µm were selected and mixed with fresh compost in equal mass ratios. Biodegradation of microplastic particles in compost was tracked by extraction and direct particle visualization. Additionally, biodegradation and mineralization of microplastics to CO_2_ in compost was monitored by aerobic respirometry performed at 45 °C. Microbial enrichments were performed to isolate strains that utilize biodegradable microplastics as a carbon source and to identify possible depolymerization products.

To test this hypothesis, we physically ground a bio-based thermoplastic polyurethane (TPU-FC1) to generate microplastics and used multiple methods to confirm rapid biodegradation and disappearance of these microplastics in home composting conditions (Fig. 1). Extraction and quantification of microplastic particles indicated that TPU-FC1 particles completely degraded within 200 days, while similar particles derived from a non-biodegradable polymer, ethyl vinyl acetate (EVA), showed no reduction of particle number in the same time frame. Tracking CO_2_ production via respirometry under identical composting conditions further confirmed biodegradation and mineralization of TPU-FC1 particles. To identify organisms responsible for this biodegradation, microbial enrichments were performed using TPU-FC1 as a sole carbon and energy source. From these enrichments, a bacterial strain belonging to the genus *Rhodococcus* was isolated that grows rapidly on TPU-FC1 alone. Feeding studies using this *Rhodococcus* strain indicated that it can depolymerize the TPU-FC1 material into the starting monomers, which can be quickly consumed by the *Rhodococcus* and other microorganisms.

## Materials and methods

### Microplastics sample generation

Ethylene Vinyl Acetate (EVA) material was purchased as 12″ × 12″ × 0.375″ solid sheets from Curbell Plastics (https://www.curbellplastics.com). Thermoplastic polyurethane (TPU) materials were prepared as described previously^12,13^ through the reaction of a linear aliphatic polyester polyol, composed solely of a bio-derived aliphatic diol and two bio-derived aliphatic diacids, with either a linear aliphatic diisocyanate (TPU-FC1) or an aromatic diisocyanate (cell phone case). Microplastics of various materials were generated by sanding using a benchtop belt sander with 80 grit sandpaper. A belt sander was chosen instead of alternative methods, such as a cryogrinder, due to its common availability, cost effectiveness, and similarity to abrasion-dependent microplastic generation that often occurs with plastic use. To prevent cross contamination between different materials, different sanding belts were used for each material. Materials were repeatedly submerged in liquid nitrogen to minimize heating and melting during the grinding procedure. Sanded microplastics were further size selected using a sieve stack and microplastics smaller than 5 mm and larger than 350 µm were retained for further experiments.

### Respirometry experiments

Biodegradation of TPU microplastics was performed under controlled composting conditions according to the ASTM 5338-15 standard, monitoring CO_2_ evolution, except for the use of home compost temperature of 45 °C instead of thermophilic temperatures to enhance relevance to non-industrial biodegradation conditions. Cellulose served as the positive control; ethylene–vinyl acetate (EVA) and compost only samples served as negative and blank controls, respectively. The experiment was stopped after 200 days of incubation, once the mineralization plateau was reached for most samples. Fresh compost was collected from Roger’s Community Garden composting site at the University of California San Diego^10^ and filtered with a 1 cm sieve. 20 g of sample microplastics material and 240 g of fresh compost were thoroughly mixed and samples were incubated in a respirometer (Echo Instruments). Sample chambers were maintained at 45 °C and at ~ 58% relative humidity for the duration of experiment. Sample chambers were opened biweekly to mix compost and add water as needed. Percent biodegradation and biodegradation relative to cellulose was calculated by the following formula:

$$\% \,theoretical \, biodegradation=\frac{{CO}_{2} \, production\left(g\right) \, sample -{ CO}_{2} \, production\left(g\right) \, compost \, only }{sample \, mass\left(g\right) \times \% \, carbon \, content \, of \, sample \times (\% \, carbon \, content\, {CO}_{2}{)}^{-1}}$$

### Microplastics extraction and quantification

Samples of compost to be extracted for microplastics were first dried in open glass beakers in a drying oven maintained at 70 °C for at least 24 h before extraction. Extraction of microplastics was performed using a previously described protocol with minor variations^15^. Briefly, 0.5 g of dried compost sample was sonicated (50% amplitude, 2 × 30 s) in distilled water and the resulting slurry was passed through several metal mesh sieves. Retained solids on the 0.5 mm and 0.35 mm mesh filters were transferred to a separatory funnel using a solution of saturated CaCl_2_ (density = 1.55 g/cm^3^). After vigorous swirling, the solids were allowed to separate based on density for 2 h at room temperature (RT). Approximately 75% of the bottom phase was drained and the remaining top layer containing microplastics was vacuum filtered onto a GF/A filter in a glass filtering apparatus. The filter containing microplastics was transferred to a glass petri dish with 30 mL of Nile Red staining solution (0.2% (w/v) Tween 20 with 4 µg/mL Nile Red)^16^ and gently mixed for 15 min. Stained particles were then vacuum filtered onto a GF/A filter and illuminated with a Blue LED transilluminator and photographed with a Canon DSLR camera. Fluorescent microplastic particles on filters were quantified with ImageJ.

### Media and growth conditions

The minimal defined media for enrichments and growth of various strains on TPU-FC1 was Brunner medium 457 (https://bacmedia.dsmz.de/medium/457). For isolation of various strains, either Luria–Bertani (LB) or Reasoner’s 2A media (R2A) was used as a rich media. For solid media, 15 g/L of agar was added to the appropriate liquid media. TPU materials were sanded and sieved into a fine powder (< 5 mm and > 350 µm particle size) as described above and autoclaved dry in borosilicate glass flasks for all microbial growth experiments. Microbial growth was quantified by OD_600_ using a spectrophotometer.

### Enrichment and identification of TPU utilizing bacteria from compost

Samples of compost (~ 0.5 g) were combined with 5 mL of sterile PBS and shaken for 1 h at RT. An aliquot of this slurry was inoculated into 50 mL of Brunner minimal media (https://bacmedia.dsmz.de/medium/457) containing shredded 1% (w/v) TPU-FC1 as a sole carbon and energy source. Cultures were maintained at RT (~ 22 °C) with shaking. Enrichment cultures were passaged once a week by 100-fold dilutions into fresh media containing TPU-FC1 and this passaging procedure was repeated five times. After the fifth passage, aliquots of the culture were diluted and plated onto LB or R2A solid media and plates were incubated at RT. Colonies of interest were restreaked three times for strain purification and subsequently grown in either LB or R2A broth as needed. Isolated strains were re-screened for their ability to catabolize TPU-FC1 as a sole carbon and energy source in minimal media as described above.

Strains of interest were grown in R2A or LB liquid as needed and genomic DNA was extracted using a Zymo Research Soil and Tissue Genomic DNA purification kit following manufacturer guidelines. For phylogenetic strain identification, the 16S rRNA gene was PCR amplified from purified genomic DNA using the universal prokaryotic 16S rRNA primer set (27F = AGAGTTTGATCMTGGCTCAG, 1492R = GGTTACCTTGTTACGACTT) using Q5 polymerase (New England Biosciences). PCR products were purified using the Promega SV Gel PCR purification kit and sent to Primordium labs for DNA sequencing. Sequences were queried by BLAST against the NCBI 16S rRNA database for phylogenetic identification.

### Identification of depolymerization products by isolated bacteria

Isolates of interest were grown with TPU-FC1 as the sole carbon source as described above. At indicated timepoints, 1 mL of culture was removed and centrifuged to remove growing cells. The cell free supernatant was filtered through a 0.2 µm (PES) filter and stored at − 20 °C until further analysis. Samples were speed vacuumed at 45 °C for 200 min to evaporate the media. 5 µL of 1 M NaOH and 50 µL of internal standard (1 µL/mL diethyl succinate in ethyl acetate) were added. Samples were extracted three times with 100 µL ethyl acetate, which was then blown off with nitrogen gas. 50 µL of ethyl acetate, 2 µL MilliQ water, and 20 µL chloroform were added to each sample. 25 µL was silylated with 25 µL *N*-Methyl-*N*-trimethylsilyltrifluoroacetamide (MSTFA) and reacted for 20 min. Samples were run on a 7820A GC/5975 MS with an Agilent J&W HP-5 column with dimensions 30 m × 0.25 mm × 0.25 µm. Samples were injected at 2 mL/min at a split of 10:1. The inlet temperature was 250 °C. The oven was at 50 °C for 5.35 min, then ramped to 120 °C at 10 °C/min, then ramped to 280 °C at 50 °C/min and held for 10 min.

### Imaging of biodegraded samples with scanning electron microscopy

Samples of interest were fixed for imaging by using a formaldehyde fixation protocol similar to previous work with slight modification^10^. Samples of interest were immersed in phosphate buffer containing 4% formalin and incubated for 2 h at RT for fixation. Fixation solution was removed and samples were washed once with an equal volume of phosphate buffer and then sequentially exposed to an ethanol gradient (50, 65, 80, 95%) for sample dehydration. Samples were then dried and attached to aluminum stubs using carbon tape, after which an Emitech K575X Sputter Coater was used to deposit an iridium layer and excess coating was removed with compressed air prior to imaging. All samples were imaged at high vacuum using an FEI Quanta FEG 250 scanning electron microscope, at magnifications ranging from 100× to 8000× magnification and visually inspected for structural modifications and the presence of microbial biofilms.

## Results and discussion

### Comparative biodegradation of microplastics

**Figure 2:**
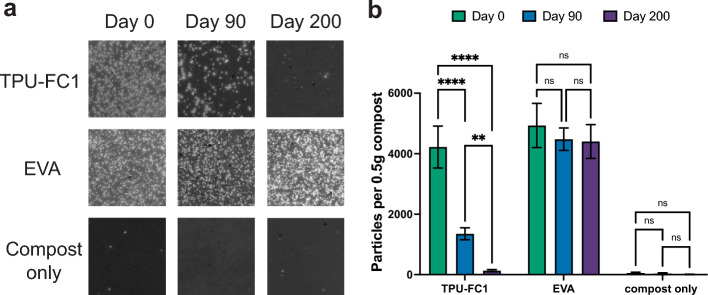
Comparison of persistant EVA and transient TPU-FC1 microplastic particles. (**a**) Representative images of microplastic extraction filters. Microplastic particles were stained with Nile Red and illuminated with blue light for imaging. Particles appear bright against the darker filter background. Full color images and images with quantified microplastics using ImageJ are provided in Supplemental Fig. 1. (**b**) Particle counts of various microplastics materials over time in compost and background compost at Day 0, 90, and 200. Values represent the average of at least 3 independent microplastic extraction procedures. Two-way ANOVA, ****p < 0.0001; **p < 0.01; ns = *p* > 0.05.

Microplastic particles generated from a bio-based polyester thermoplastic polyurethane (TPU-FC1), and from a petroleum-based thermoplastic ethyl vinyl acetate (EVA), were mixed with freshly collected compost in a defined 1:12 mass ratio and incubated in glass dishes at 45 °C. Samples of the compost containing the microplastics were removed at 0, 90, and 200 days, and a differential density-based extraction procedure was performed to separate microplastic particles from the compost matrix. Microplastic particles, visualized by fluorescent staining with Nile Red, were abundant at the day zero time point in both plastic-containing samples, while few particles were observed in the compost control, as expected (Fig. 2). It is important to note that though the blank sample has significantly fewer microplastic particles than the plastic-containing samples across all time points, the number observed was non-zero (52 ± 29 on Day 0), consistent with findings of microplastics in nearly all environments^1,2,4^. Examination of samples after 90 days of aerobic composting showed a 68% decrease in the number of particles for TPU-FC1, from 4221 ± 694.7 to 1352 ± 195.9 particles per half gram of compost (Fig. 2b). In contrast, the amount of EVA particles remaining after 90 days were not significantly different from the initial time point. After 200 days of aerobic composting, particle counts of microplastics in the TPU-FC1 sample dropped to 135 ± 34 particles per half gram of compost, a 97% overall reduction from the starting count. In contrast, particle counts of the EVA microplastics, a non-biodegradable polymer, remained unchanged at the 200-day time point (Fig. 2b).

Extraction and direct visualization of microplastics indicated that we successfully generated microplastics and were able to accurately extract and quantify particles above the 350 µm mesh size threshold. The loss of TPU-FC1 microplastic particles is indicative of biodegradation and that microplastics generated from this material are transient and not persistent, unlike the persistence of EVA microplastics that is consistent with EVA not being biodegradable. While this extraction and visualization procedure can only visualize particles within a particular size range (> 350 µm), we expect that smaller particles would degrade at similar or perhaps faster rates given their larger surface area to volume ratio, with the fate of the carbon in those degraded microplastics being biochemically turned into biomass and CO_2_ through mineralization.

### Tracking biodegradation of TPU-FC1 microplastics by respirometry

**Figure 3:**
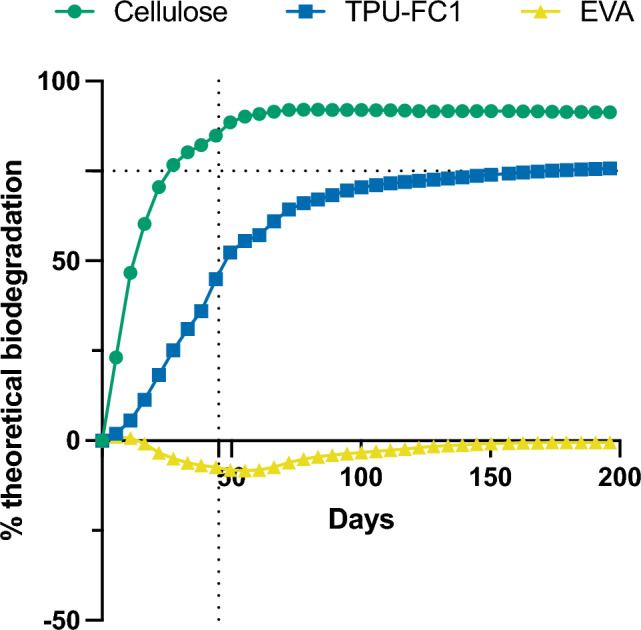
Biodegradation of materials in compost monitored by CO_2_ evolution respirometry. Percent theoretical biodegradation calculated as described in Materials and Methods. Dashed lines indicate 75% theoretical biodegradation at 45 days, which the cellulose control material must reach as an experimental validation condition of the ASTM5338 standard.

To verify the biodegradation of these materials as microplastic particles and their predicted fate due to complete mineralization, a parallel set of samples with identical microplastics and compost were incubated at 45 °C in respirometry chambers designed to track CO_2_ evolution under aerobic conditions consistent with the ASTM 5338-15 standard method (Fig. 1). Additional compost only and cellulose samples were also included as internal controls to monitor background CO_2_ evolution and bioactivity of compost on a control substrate, respectively. As shown in Fig. 3, the cellulose positive control reached 75% CO_2_ evolution within 45 days, indicating that the compost inoculum was sufficiently active as required by the ASTM 5338 standard. As expected for a non-biodegradable material, EVA microplastic particles showed no CO_2_ evolution over the course of the 200-day experiment. Instead, negative biodegradation values were calculated as a consequence of subtraction of the blank compost’s CO_2_ evolution, indicating that the EVA either absorbs CO_2_ from the metabolically active compost or the EVA microplastics kill the metabolic activity of the compost’s microbiome. In close agreement with the extraction and visualization particle count data, the bio-based TPU-FC1 transient microplastics displayed significant biodegradation, reaching 76% CO_2_ evolution at the 200-day time point. Thus, respirometry confirms the biodegradability of the bio-based TPU-FC1 material and demonstrates that one outcome of that biodegradation is the conversion of the carbon from the microplastics into CO_2_.

### Isolation and characterization of bacteria capable of biodegrading TPU-FC1 material

Microbial enrichments were performed to confirm the fate of TPU-FC1 as biomass, identify microbial species capable of degrading the bio-based TPU-FC1 material, and identify intermediate depolymerization products. Minimal media containing shredded TPU-FC1 as the sole carbon source was inoculated with samples of compost that showed active biodegradation activity. Cultures were diluted 100-fold every week for five weeks and dilutions plated onto either LB or R2A solid agar plates. Colonies were picked based on unique morphological characteristics and restreaked multiple times for clonal isolation. Axenic isolates from the 5-week time point were screened for their ability to utilize TPU-FC1 as a sole carbon source for growth as monitored by optical density, and confirmed strains were identified by 16S rRNA sequencing. A previous culture collection derived from enrichments using various biodegradable polyurethane foams^10^ was also screened for strains capable of using TPU-FC1 as a sole carbon source. It should be noted that this enrichment and screening strategy is inherently selective for those microbes that can consume TPU-FC1 and also grow under laboratory culturing conditions. Thus, the isolates recovered may not represent the entire diversity of bacteria or fungi capable of degrading and mineralizing TPU-FC1, but do confirm the existence of such microbes and enabled the biodegradation characterization experiments described below.

**Figure 4:**
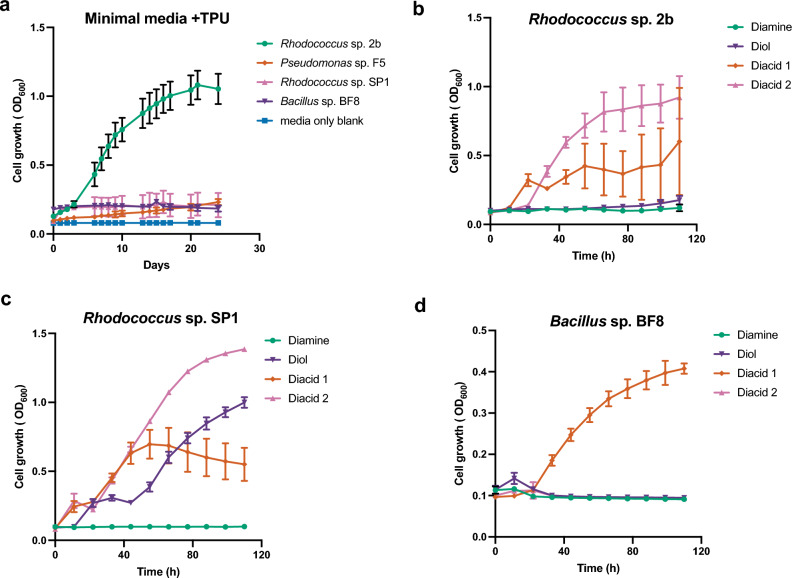
Characterization of bacteria capable of utilizing thermoplastic polyurethane as a sole carbon source. (**a**) Growth curves of various strains grown with TPU-FC1 as a sole carbon source in minimal media at 22 °C. (**b**–**d**) Growth of various strains on diacids, diol, and diamine monomers derived from the TPU-FC1 formulation, each compound was provided as sole carbon sources in minimal media at a concentration of 1 g/L. Data presented are the average of three biological replicates and error bars represent standard deviations. Note the change in y-axis scale for Panel D.

Two novel bacterial strains capable of utilizing TPU-FC1 as a sole carbon source were isolated from this set of enrichment experiments (Table 1, Fig. 4a). The best performing strain, designated 2b, was identified as a member of the *Rhodococcus* genus based on 16S rRNA sequence. Another strain, BF8, isolated in these enrichments was identified as a member of the genus *Bacillus*. From our previous terrestrial culture collection, we noted that two strains, *Rhodococcus* sp. SP1 and *Pseudomonas* sp. F5, also demonstrated some ability to catabolize TPU-FC1 under these experimental conditions (Fig. 4a). Bacteria belonging to the genus *Rhodococcus, Pseudomonas,* and *Bacillus* have been previously demonstrated to be involved with degradation of polyurethane^10,17–22^. Their relative ubiquity in soil and other terrestrial environments with high biodegradation activities strongly suggests that TPU-FC1 would likely biodegrade in other environments besides compost.

**Table 1:** Strains capable of catabolizing TPU-FC1.

Strain	Closest phylogenetic match based on 16S rRNA sequence (% nucleotide ID)	Source
2b	*Rhodococcus fascians* strain CF17 (99.5%)	This study
SP1	*Rhodococcus qingshengii* JCM 15,477 (100%)	^10^
F5	*Pseudomonas aeruginosa* DSM 50,071 (100%)	^10^
BF8	*Bacillus altitudinis* 41KF2b (100%)	This study

Based on the monomer composition of TPU-FC1, generated from two diacids, one diol, and a linear aliphatic diisocyanate that is predicted to be released as a diamine through the biodegradation process, these isolated strains were assayed for their ability to use each monomer component as a sole carbon source in minimal media. Growth with either of TPU-FC1’s diacids as sole carbon sources was relatively robust across all three strains. While *Rhodococcus* sp. 2b grew well on TPU-FC1 as a sole carbon source, it grew relatively modestly and with high variability on the various monomers except for diacid 2 (Fig. 4b). In contrast, while growth of *Rhodococcus* sp. SP1 on the TPU-FC1 polymer was relatively modest (Fig. 4a), growth on both diacids and diol components was relatively robust compared to the other strains (Fig. 4c). These observations indicate that some strains may be more capable of cleaving TPU-FC1 into monomers, while others are more adept at scavenging the released monomers, supporting the notion that a community of organisms, rather than a single organism, enables efficient biodegradation in natural environments. Growth with the aliphatic diamine as a sole carbon source was nearly undetectable across all strains, though this could indicate that the diamine is not the correct product of biodegradation of the isocyanate moiety. Based on the robust growth characteristics of *Rhodococcus* sp. 2b on TPU-FC1, it was selected for further investigation of possible depolymerization products.

### Identification of TPU-FC1 depolymerization products derived from *Rhodococcus* sp. 2b

**Figure 5:**
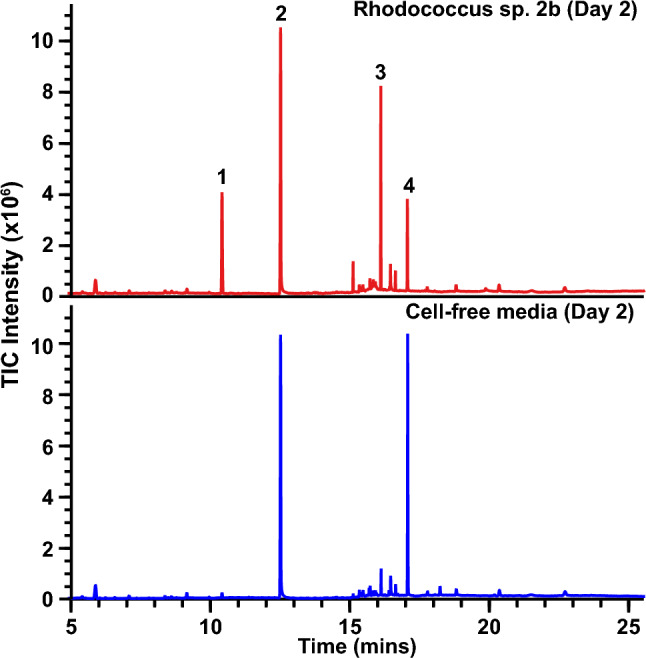
Depolymerization of TPU-FC1 into monomers and oligomers. GC chromatograms of cell-free supernatants from a 2-day old culture of *Rhodococcus* sp. 2b grown with TPU-FC1 as a sole carbon source (top) and a cell-free media control (bottom). Peaks 1 and 3 appeared only in the presence of *Rhodococcus* after 2 days incubation. Peak 2 is the diethyl succinate internal standard. Peak 4 was present in both the sample and control and was assumed to be a component of the media. Peak 1 was identified as the TPU-FC1 diol monomer based on its fragmentation pattern and compared against an authentic standard. Peak 3 was proposed to be a TPU-FC1 oligomer based on fragmentation structure prediction.

Given the robust growth of *Rhodococcus* sp. 2b using TPU-FC1 as a sole carbon source, further analysis of culture supernatants were performed to identify intermediate depolymerization products. Culture supernatants taken after 5 days of growth did not show the presence of any predicted monomers, in contrast to the detectable presence of monomers in a cell-free control sample of minimal media with TPU-FC1. This indicated that the *Rhodococcus* efficiently consumes released monomers and that an earlier time point was required to capture intermediate depolymerization products. GC–MS analysis of *Rhodococcus* sp. 2b supernatants taken after only two days of growth on TPU-FC1 as a sole carbon source showed significant accumulation of the diol monomer (Peak 1) and a TPU oligomer (Peak 3) in the culture supernatant, neither of which were present in cell-free control supernatant after two days of incubation (Fig. 5). Based on mass spectral data, the oligomer was predicted to contain a single urethane bond linking an aliphatic diamine to a linear diacid. The release and accumulation of these monomers indicates that *Rhodococcus* sp. 2b can depolymerize the supplied TPU-FC1 material. The lack of signals for diacid components in the TPU-FC1 formulation can be explained by this strain’s ability to utilize these compounds as carbon sources (Fig. 4b). Furthermore, the accumulation of diol agrees with this strain’s reduced ability to grow with the diol compound as a sole carbon source.

These results are consistent with depolymerization of the TPU-FC1 polymer and enzymatic cleavage of urethane and/or ester bonds found therein. While the newly isolated *Rhodococcus* sp. 2b strain is unable to completely catabolize the complete suite of monomers derived from this polymer as sole carbon sources, it is expected that other members of a given microbial community in the environment could catabolize the remaining monomeric and/or oligomeric components. Interestingly the TPU oligomer noted in the culture supernatant after two days was no longer detected at later time points, especially at higher cell densities. Given the presence of a urethane bond in the oligomer, it is suspected that a urethane bond cleaving enzyme produced by *Rhodococcus* sp. 2b cleaves this bond to further catabolize the remaining compound. Previous work with other polyester-polyurethane degrading bacteria has indicated that depolymerization can occur in a stepwise fashion with ester bonds being initially hydrolyzed followed by urethane bonds^23–25^. Furthermore, a urethane-bond cleaving enzyme found in *Rhodococcus equi* TB-60 was found to be inducible by the presence of amide bond containing compounds^17^. Although this data suggests a specific degradation pathway common with previous literature, other pathways may exist when in the presence of microbes other than *Rhodococcus* sp. 2b or alternative volatile byproducts may have been generated that are not observable here due to the ethyl acetate removal step during sample preparation.

### Potential product applications of biobased TPU materials

Given the conclusion that the bio-based TPU-FC1 is fully biodegradable, we explored possible product applications for this material and investigated whether biodegradation of these products occurs in a manner similar to the generated microplastics. Softer TPU’s have a well-documented application in fabric coating for the purpose of water-proofing and/or sealing. TPU materials can also be injection molded to form plastic products, such as phone cases. To test the biodegradation of these TPUs in a plausible real-world application we generated TPU-FC-coated cotton fabrics and an injection molded phone case using a similar TPU formulation that uses the same polyol but an aromatic isocyanate in place of TPU-FC1’s aliphatic isocyanate and incubated these materials in compost under identical conditions as the microplastics assays described above. As shown in Supplemental Fig. 2, degradation of these various materials was evident at the macro scale compared to their respective non-composted controls (Supplemental Fig. 2). The coated fabrics (Supplemental Fig. 2A, B) biodegraded rapidly, leaving behind fabric which fragmented easily upon retrieval from compost after only two weeks in compost (Supplemental Fig. 2B). In contrast, the control TPU-coated fabrics that were not composted remained intact and resistant to tearing. After a year of compost incubation, the injection molded phone case became brittle and showed signs of discoloration, biofilm development, cracking, and structural degradation (Supplemental Fig. 2C) compared to the phone case prior to incubation in compost (Supplemental Fig. 2D).

**Figure 6:**
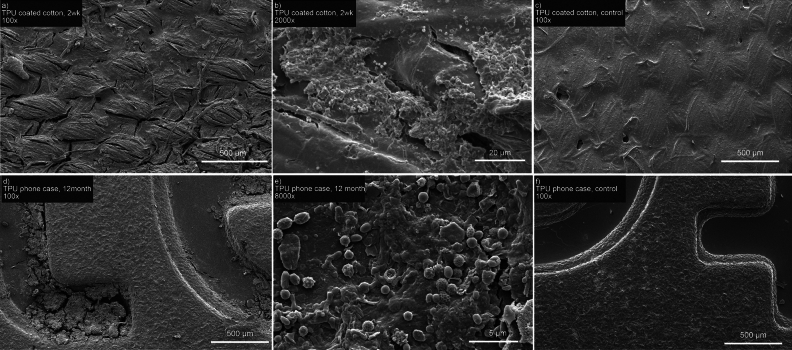
Scanning electron microscopy of biodegraded TPU products compared to controls. (**a**,**b**) TPU-coated fabric after 2-week incubation in compost. (**c**) Control image of TPU-coated fabric that was not placed in compost. (**d**,**e**) Injection molded TPU phone case after 12-month incubation in compost. (**f**) Control image of injection molded TPU phone case prior to placement in compost.

Scanning electron microscopy further confirmed the biodegradation and overall structural changes in these products. While the control TPU-coated fabrics that were not composted show the presence of the TPU coating masking the underlying fibrous cotton material (Fig. 6c), the TPU-coated fabrics composted for two weeks, which were cut from the same larger piece of TPU-coated fabric as the control samples in Fig. 6b and Supplemental Fig. 2B, showed clear loss of the TPU coating, revealing the underlying fabric (Fig. 6a). The coating that remained also showed significant cracking and degradation, along with the accumulation of microbial biofilms (Fig. 6b). Similar results were noted for the injection molded phone case, which showed similar signs of cracking and fissure formation (Fig. 6d) along the surface of the material after 12 months in compost that are not present on the surface of the material prior to compost incubation (Fig. 6f). As with the coated fabrics, microbial biofilms were readily observed on the surface of the TPU material (Fig. 6e).

## Conclusion

In this work, particle count and respirometry experiments demonstrated that microplastic particles from a bio-based thermoplastic polyurethane can rapidly biodegrade and therefore are transiently present in the environment. In contrast, microplastic particles from a widely used commercial thermoplastic, ethyl vinyl acetate, persists in the environment and showed no significant signs of biodegradation over the course of this experiment. Bacteria capable of utilizing TPU-FC1 as a carbon source were isolated and depolymerization of the material was confirmed by the early accumulation of monomers derived from the original polymer, which are metabolized by microbes in short order. Finally, we demonstrated that prototype products made from these materials biodegrade under home compost conditions. The generation of microplastics is an unavoidable consequence of plastic usage and mitigating the persistence of these particles by adoption of biodegradable material alternatives is a viable option for a future green circular economy.

### Supplementary Information

Supplementary Figures.

## Data Availability

The data that support the findings of this study are provided within the manuscript or in the supplementary material of this article.
