# Supplementary material for: Rapid biodegradation of microplastics generated from bio-based thermoplastic polyurethane
Source: Sci Rep. 2024 Mar 12;14:6036. doi: 10.1038/s41598-024-56492-6 (PMC10933395; doi:10.1038/s41598-024-56492-6)
Supplement: Supplementary file 1 — Supplementary Figures. [file 41598_2024_56492_MOESM1_ESM.pdf]

A) TPU-FC1

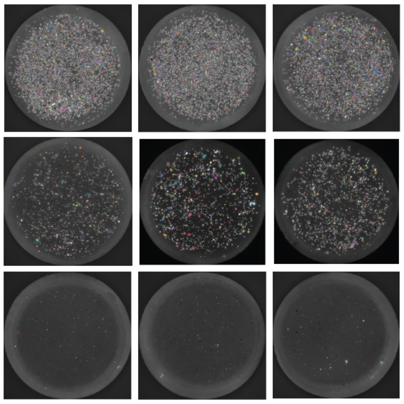

	TPU-FC1
Day 0	3536
Day 0	4203
Day 0	4925
Day 90	1457
Day 90	1126
Day 90	1473
Day 200	151
Day 200	157
Day 200	96

B) EVA

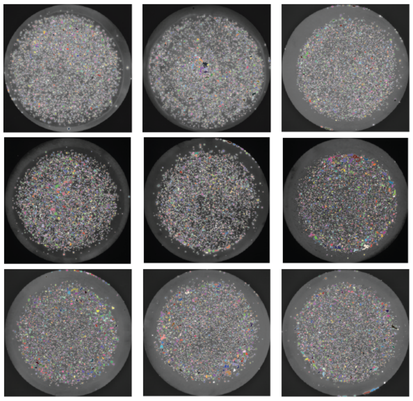

	EVA
Day 0	4339
Day 0	5750
Day 0	4712
Day 90	4053
Day 90	4731
Day 90	4656
Day 200	4578
Day 200	4855
Day 200	3779

C) Compost only

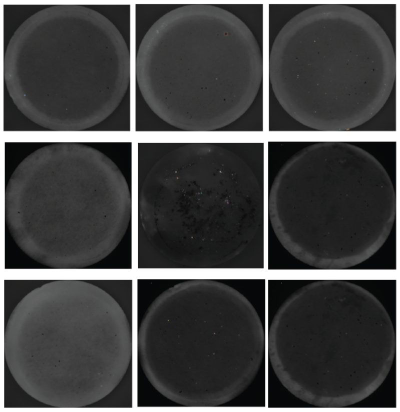

	compost only
Day 0	51
Day 0	23
Day 0	81
Day 90	7
Day 90	68
Day 90	6
Day 200	9
Day 200	19
Day 200	5

Supplemental Figure 1- Full scale images of all microplastic extraction filters. Tables to the left are particle counts of quantified microplastics generated in ImageJ. (A) Filters from TPU-FC1 at the indicated times/replicates. (B) Filters from the EVA samples at the indicated times/replicates. (C) Compost only control filters at the indicated times/replicates.

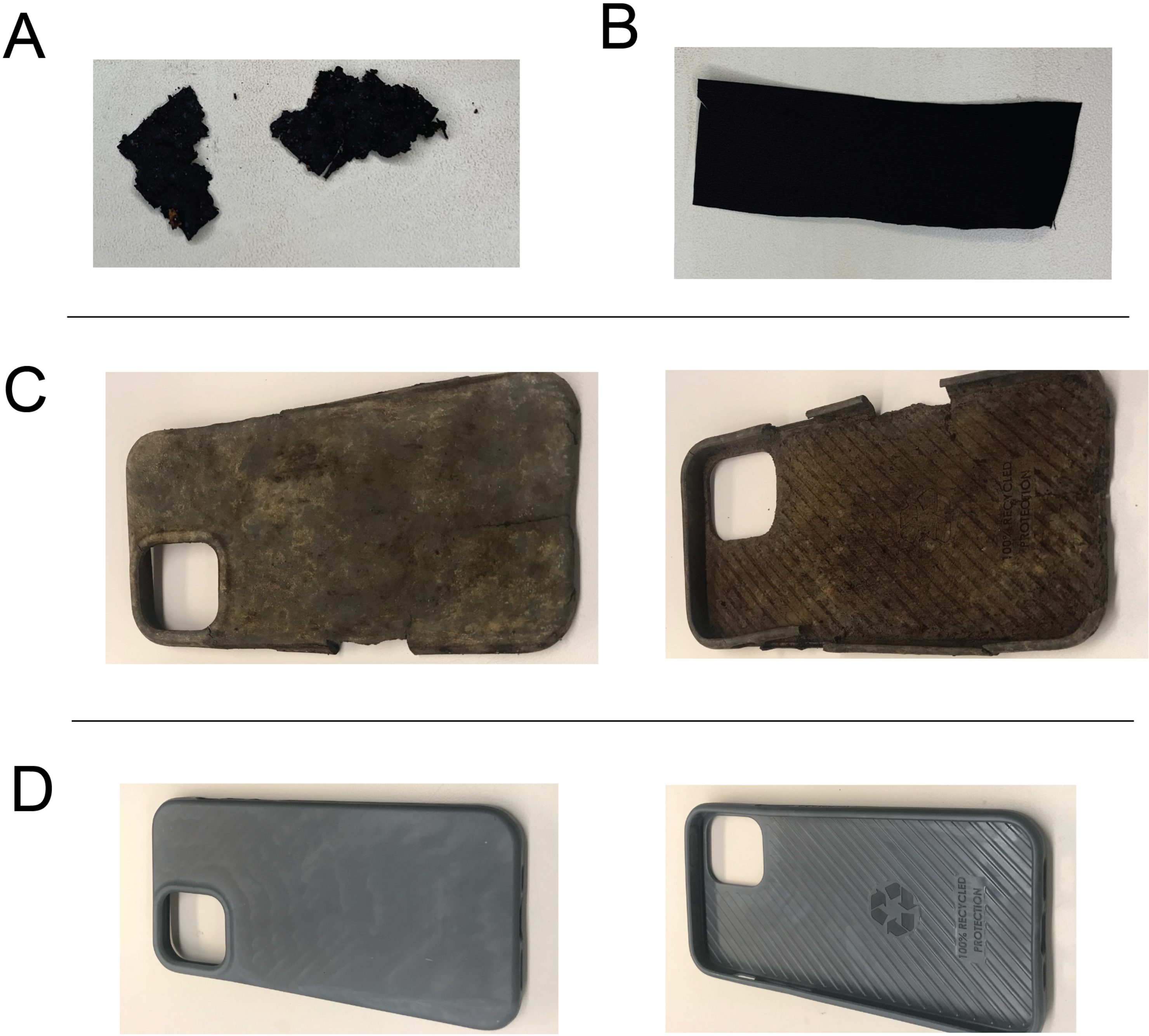

Supplemental Figure 2- Biodegradation of various prototype products made from bio-based TPU materials. (A) TPU-coated fabric after 2wk in compost conditions. (B) Control sample of TPU-coated fabric that was not exposed to compost after 2wk. (C) Injection molded TPU phone case after one year in compost. (D) Control images of TPU phone case not exposed to compost.
